# CCR6 as a Potential Target for Therapeutic Antibodies for the Treatment of Inflammatory Diseases

**DOI:** 10.3390/antib12020030

**Published:** 2023-04-20

**Authors:** Sara Gómez-Melero, Javier Caballero-Villarraso

**Affiliations:** 1 Maimonides Biomedical Research Institute of Cordoba, Avda. Menéndez Pidal s/n, 14004 Córdoba, Spain; 2 Department of Biochemistry and Molecular Biology, Faculty of Medicine and Nursing, University of Córdoba, Avda. Menéndez Pidal s/n, 14004 Córdoba, Spain

**Keywords:** CCR6, antibody, therapy, GPCRs, inflammation, immune system, Th17 cells

## Abstract

The CC chemokine receptor 6 (CCR6) is a G protein-coupled receptor (GPCR) involved in a wide range of biological processes. When CCR6 binds to its sole ligand CCL20, a signaling network is produced. This pathway is implicated in mechanisms related to many diseases, such as cancer, psoriasis, multiple sclerosis, HIV infection or rheumatoid arthritis. The CCR6/CCL20 axis plays a fundamental role in immune homeostasis and activation. Th17 cells express the CCR6 receptor and inflammatory cytokines, including IL-17, IL-21 and IL-22, which are involved in the spread of inflammatory response. The CCL20/CCR6 mechanism plays a crucial role in the recruitment of these pro-inflammatory cells to local tissues. To date, there are no drugs against CCR6 approved, and the development of small molecules against CCR6 is complicated due to the difficulty in screenings. This review highlights the potential as a therapeutic target of the CCR6 receptor in numerous diseases and the importance of the development of antibodies against CCR6 that could be a promising alternative to small molecules in the treatment of CCR6/CCL20 axis-related pathologies.

## 1. Introduction

G protein-coupled receptors (GPCRs) are one of the most abundant receptors encoded in the human genome, with over 800 members, and transmit approximately 80% of signal transduction across cell membranes [1,2]. GPCRs signal through activation of Gα, Gβ and Gγ subunits of heterotrimeric G protein and β-arrestin protein mediators [3]. GPCRs are involved in a broad range of key biological processes, including homeostasis, proliferation and chemotaxis of cells, and have been implicated in a considerable number of diseases, such as cancer, inflammation and infection [4,5].

Chemokine receptors are a family of GPCRs regulated by small ligands known as chemokines. These molecules are low molecular weight proteins with a globular core structure stabilized by 1–2 conserved disulfide bridges essential in leukocyte trafficking through the formation of chemotactic gradients [6,7,8]. Chemokines and chemokine receptors play important roles in a broad range of biological and pathological processes controlling the activation, migration, differentiation and survival of leukocytes and other hematopoietic cells [9,10]. 

The CC chemokine receptor 6 (CCR6) is a class A GPCR belonging to the chemokine family and is recognized for its invaluable therapeutic potential in research related to the immune system [11]. CCR6 is expressed in numerous cells, including B cells, immature dendritic cells (DCs), innate lymphoid cells (ILCs), Langerhans cells, neutrophils, regulatory T (Treg) cells and T helper 17 (Th17) cells [12,13]. The only chemokine ligand of CCR6 is CCL20 and, in humans and mice, is expressed by neutrophils, Th17 cells and peripheral blood mononuclear cells [8,14,15]. This axis plays exclusive roles in immune homeostasis and activation. The influence of the CCR6/CCL20 partnership via a pleiotropic immune mechanism in the respiratory, nervous, excretory, skeletal, gastrointestinal and reproductive systems has been demonstrated, manifesting as numerous diseases [11].

Although the relationship between CCR6 and many diseases has been widely studied, at this moment, there is no therapeutic agent against CCR6 approved [16]. Given the important roles that CCR6 and CCL20 play in clinical pathophysiology, this axis is considered a potential therapeutic target. An antagonizing monoclonal antibody could be a potential alternative to conventional small-molecule drugs and an effective strategy for treating certain inflammatory and autoimmune diseases. This review recapitulates the role of CCR6 in human pathologies and the use of anti-CCR6 antibodies as a potential therapeutic target for diseases associated with the CCR6/CCL20 axis. 

## 2. Description of CCR6 

### 2.1. Biochemical Characteristics and Structure

The human CCR6 gene was mapped to chromosome 6q27, outside the main cluster of CC chemokine receptor genes on chromosome 3p [17]. The CCR6 receptor is a protein embedded in the cell membrane with seven transmembrane α-helices connected by three extracellular loops (ECL1–3) and three intracellular loops (ICL1–3). The extracellular (EC) region, which is responsible for ligand binding, also contains the N-terminus and the intracellular (IC) region, which includes cytoplasmic helix H8 and a C-terminus, interacts with G proteins, arrestins and other downstream effectors [18].

The only known high-affinity chemokine ligand of the CCR6 receptor is CCL20; however, low-affinity binding of the human beta-defensins (hBDs)-1 and -2, a group of anti-bacterial peptides, to CCR6, has been reported [6]. Some chemokines bind to more than one receptor, but CCL20 binds specifically to the CCR6 receptor and forms an exclusive, monogamous pair [19]. The binding site of CCL20 to CCR6 is a shallow extracellular pocket, in contrast to the deep agonist-binding sites observed in other class A GPCRs, producing interactions with the three ECLs. Moreover, the N-terminal residues Y27 and L38 from the receptor wrap onto the globular core of CCL20, serving as another critical docking of chemokine binding [8].

**Figure 1:**
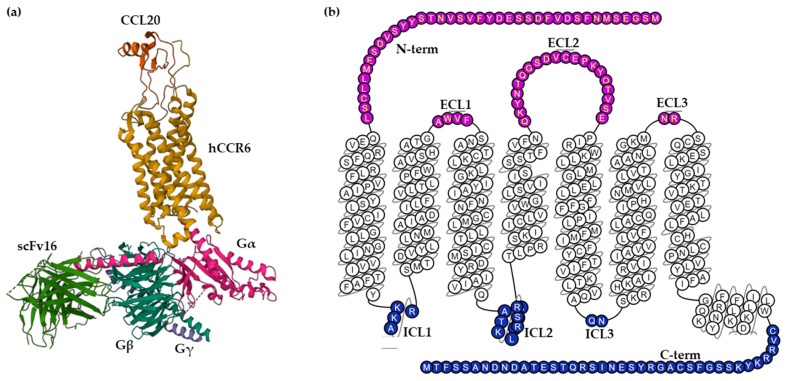
(**a**) Cryo-EM structure of the human chemokine receptor CCR6 in complex with CCL20 and a Go protein (PDB ID: 6WWZ) colored by subunit. Wasilko et al. introduced the single-chain variable fragment (scFv16) in the complex to slow down the dissociation. (**b**) Snake helix box diagram depicts human CCR6 topology as seen from the side (GPCR database). Extracellular domains: purple; transmembrane domains: white; intracellular domains: blue.

CCR6 is composed of 139,737 bases long and encodes a protein with 374 amino acids with a molecular weight of 42 kDa [20]. Wasilko et al. elucidated a cryo-electron microscopy (Cryo-EM) structure of human CCR6 receptor bound to CCL20 and Go protein (Figure 1), giving important insights into the mechanism of activation of CCR6 [8]. Furthermore, a homology model of human CCR6 is available in the GPCR database (GPCRdb). Snake and helix box diagrams are 2D receptor topology accessible plots that map the position of binding residues as seen from the extracellular and membrane sides, respectively [21].

### 2.2. Expression of CCR6

Human tissue expression of CCR6 can be predominantly seen in the appendix, pancreas, lymph nodes, spleen and, with lesser expression, in the fetal liver, testis, colon, small intestine and thymus [13,22]. 

At the cellular level, CCR6 is expressed in a variety of immune cell types (Table 1), consistent with its well-established role in inflammation. There are numerous leukocyte cohorts such as B cells, dendritic cells, innate lymphoid cells 3 (ILC-3), T cells, specifically pro-inflammatory Th17 cells and immune regulatory Treg cells, and neutrophils in which CCR6 is upregulated [19,22,23,24,25,26,27,28,29,30].

CCR6 is found in various B cell subtypes [13]. The receptor is expressed at the cell surface of circulating, naïve and memory but not germinal center B cells. On neutrophils, it has been reported that CCR6 can be upregulated after treatment with cytokines in vitro [24]. ILC-3 cells and immature DCs also expressed CCR6, although its expression on immature DC is lost following their maturation [25]. Moreover is known that CCR6 is expressed on multiple DCs subsets, including Langerhans cells [26].

Natural killer T (NKT) cells are a T cell subset that expresses natural killer (NK) cell markers and represent about 0.1% of peripheral blood lymphocytes. Both cells were shown to express CCR6 and be attracted by CCL20 [17]. CCR6 was found to be more frequently present in CD4^+^ T cells than in CD8^+^ T cells [17]. The CCR6 receptor plays a pivotal role in the regulation of T cells to inflammatory sites, and these cells are recruited by CCL20 interaction with CCR6 [28]. 

Quantitative information provided by The Human Protein Atlas of RNA expression across single cell types confirms the expression of CCR6 in cells shown in Table 1. The normalized single-cell RNA (nTPM) values for CCR6 are 13.5 in T cells, 15.9 in B cells, 1.2 in plasma cells, 1.4 in NK cells, 0.1 in monocytes, 0.4 in macrophages, 0.4 in dendritic cells and 0.8 in Langerhans cells [31]. The reciprocal data of normalized single-cell RNA (nTPM) for CCL20 are 46.6 in T cells, 2.1 in B cells, 3.3 in plasma cells, 1.9 in NK cells, 1275.2 in monocytes, 214.7 in macrophages, 15.2 in dendritic cells and 210.7 in Langerhans cells [32]. The data indicates a high correlation between the expression of CCR6 and CCL20 in T cells.

### 2.3. Signaling Pathways of CCR6

Activation of CCR6 elicits a combination of responses, including activation of G proteins and β-arrestins mediated signal transduction, and both have different non-overlapping functions and play important roles in CCR6 signaling [33]. 

When G proteins are activated, they dissociate from the receptor, and their α- and βγ-subunits separate and trigger the activation of a second messenger signaling and multiple intracellular pathways [34]. Upon binding the ligand to CCR6, the receptor signaling is predominantly via Gαi proteins which inhibit the cyclic 3′,5′-cyclic monophosphate (cAMP)-dependent pathway through the inhibition of adenylate cyclase [8]. Additionally, activation of CCR6 induces the release of the Gβγ subunit from the Gα subunit, which activates phospholipase C (PLC) and triggers an increase in intracellular Ca^2+^ levels [35]. When CCL20 binds to CCR6, it initiates the activation of calcium mobilization, phospholipase and phophatidyinositol-3-kinase, followed by ERK1/2 phosphorylation and actin polymerization [19].

Furthermore, like most chemokine receptors, CCR6 can interact with additional effectors such as β-arrestin. This activation involves CCR6 phosphorylation by GPCR kinases (GRK) and the β-arrestin recruitment causing receptor desensitization and internalization [7,36]. The complex CCR6–arrestin acts as a scaffold facilitating different signaling pathways through c-Src, ERK 1/2, p38, JNK or Akt, initiating a G protein-independent wave of downstream signaling [36,37,38].

CCR6 can be activated by the union of the high-affinity ligand, the CCL20 chemokine, or human β-defensins that have been reported as non-chemokine low-affinity ligands of CCR6 [14,17]. The ligand binds to the receptor recognition site formed by the turn between β2- and β3-strands and the N-terminal loop. The N-terminus of CCL20 makes key contacts with the transmembrane bundle of CCR6 for stimulating intracellular signaling and receptor activation [39,40]. The signaling by the CCR6/CCL20 axis is critical for humoral immune responses and plays an important role in the migration of Th17 cells to inflamed sites. The signaling of CCR6 by hBDs interaction is able to induce chemotaxis in immature dendritic cells and memory T cells, but its activity remains to be investigated [41,42].

## 3. The Role of CCR6 in the Immune Response

**Figure 2:**
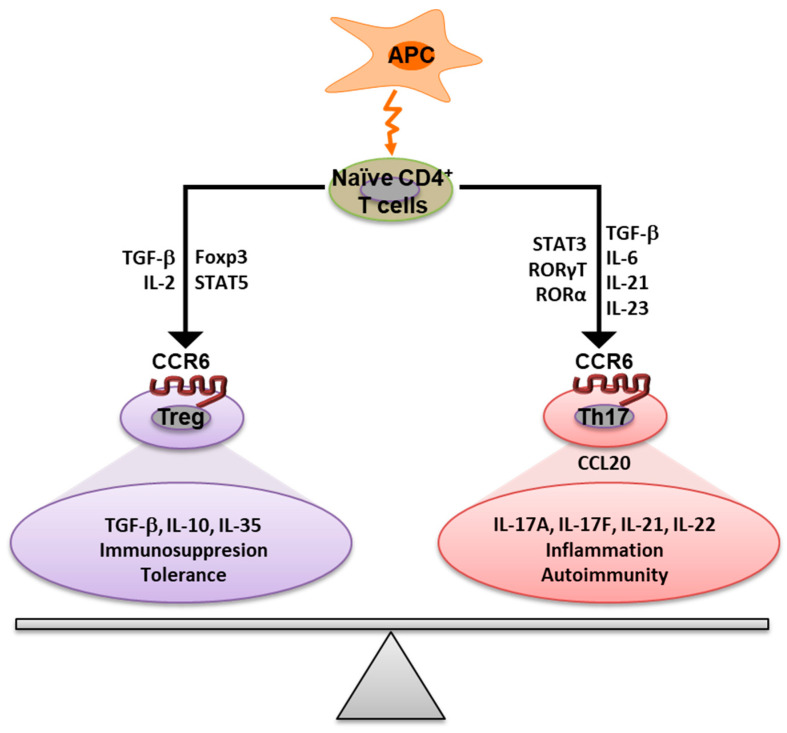
The role of the CCR6 receptor in the immune response. Antigen-specific activation of naïve CD4^+^ T cells leads to the generation of Th17 and Treg cells mediated by the cytokine milieu and the transcription factors. Both cell types express CCR6 and have opposing functions in the immune system. The Th17/Treg balance plays a critically important role in many diseases. The CCR6 receptor is important to balance the pro-inflammatory, Th17, and anti-inflammatory, Treg, cell migration mediated by the CCL20 ligand secreted by Th17 cells. APC—antigen-presenting cells.

CCR6 is recognized for its important role in immunity. The CCR6/CCL20 axis has two major roles in the immune system: immunological homeostasis and inflammation [13]. The role of CCR6 includes the balance of immune system integrity (Figure 2).

Upon activation by antigen-presenting cells (APC), naïve T cells differentiate into several subsets of effector cells, including Th17 and Treg cells [43]. The pro-inflammatory Th17 cells and the regulatory Treg cells express CCR6 on their surface and have opposing functions, primarily attributable to their cytokine profiles. Both cell types share a signaling pathway mediated by TGF-β; however, the network of cytokines and transcription factors present during cell activation regulates the differentiation into Th17 or Treg cells [44]. Th17 cells are a subset of CD4^+^ T cells that are induced upon naïve T cells during antigen priming in the presence of transforming growth factor β1 (TGF-β1), interleukin-23 (IL-23), IL-21 and IL-6 and their generation implicates the transcription factors RORγt, RORα and STAT3. Th17 cells produce pro-inflammatory cytokines such as IL-17A, IL-17F, IL-21 and IL-22 [45,46]. Treg cells are a CD4^+^ T cell subset generated under the influence of IL-2 and TGF-β and are characterized by the expression of STAT5 and the nuclear factor Foxp3 [44,47]. Treg cells produce cytokines IL-35, IL-10 and TGF-β that have anti-inflammatory functions [48].

The expression of CCR6 in both cell types is critical for maintaining immune homeostasis. While Th17 induces tissue damage and disease by releasing inflammatory cytokines, Treg cells promote immune tolerance by releasing inflammation-suppressive cytokines aiding tissue restitution [22]. CCL20 is expressed by Th17 cells but not by Treg cells, and through the interaction of CCL20 with the CCR6 receptor, Th17 cells promote the migration of Th17 and Treg cells into CCL20-enriched inflamed tissues [12,49]. CCR6 has a key role in orchestrating the migration of immune cells to inflammatory sites, and this disruption triggers the recruitment of Th17 or Treg cells to the site of infection or injury [11].

The upregulated expression of CCR6 is a critical factor that determines the development of Th17 and Treg subsets [19]. The imbalance between Th17 and Treg cells, in favor of pro-inflammatory Th17 cells or its immune regulatory partner, the Treg cells, plays a critical role in the development of immune diseases and an increased Th17/Treg ratio in patients with psoriasis, rheumatoid arthritis, multiple sclerosis and inflammatory bowel disease has been reported [43,48].

## 4. Diseases Associated with CCR6

Many reports have indicated that the CCR6 receptor plays an important role in the pathophysiology of numerous diseases, such as cancer, inflammatory diseases and autoimmune diseases (Table 2).

### 4.1. Lung Diseases

It has been described in a mouse model that the incidence of allergic asthma may be related to a remarkable increase of CCR6^+^ cells secreting IL-17 in lung tissue. This interleukin could be associated with the pathology of disease by promoting Th17 cell responses [50]. Another study suggests a role for CCR6 in the recruitment of inflammatory bronchoalveolar T cells during allergic asthmatic response [51]. Moreover, in chronic obstructive pulmonary disease (COPD), a disease characterized by chronic airway inflammation, the CCR6/CCL20 axis provides a possible mechanism for the accumulation of dendritic cells in the lungs of patients with COPD. These cells, which express CCR6, are chemoattracted by the elevated CCL20 in COPD lungs, and the accumulation of these cells increases disease severity [70,71]. 

### 4.2. Cancer

CCR6, like other chemokine receptors, induces cell migration toward a concentration gradient of the cognate chemokine ligand, CCL20, and plays a critical role in the cancer cell invasion of the lymphatic system and its spreading via blood, as well as determining the location of metastatic growth of various tumors [59]. CCR6 has been associated with a wide range of cancer types, such as hepatocellular carcinoma [61,62], colorectal cancer [63,64], breast cancer [65], prostate cancer [66], ovarian cancer [55], lung cancer [56], cancer pancreatic [57], cervical cancer [58] and renal [54]. CCL20 expression is upregulated in many cancers, such as breast cancer, hepatocellular carcinoma and pancreatic cancer [59]. CCL20 promotes the attraction of CCR6^+^ T cells to tumor sites expressing CCL20 and CCR6^+^ cancer cells to metastatic sites with abundant CCL20 [23].

### 4.3. Liver Diseases

Although the role of CCR6 in liver diseases is not clear, the association between CCR6 hepatic expression and cirrhosis and its correlation with clinical scores of disease severity has been shown [100]. Shimizu et al. describe that the receptor CCR6 and its ligand CCL20 are involved in the amplification of local immune response to inflamed livers by attracting CCR6^+^ T cells [101]. In patients with cholestatic liver diseases, the migration of Th17 cells to inflamed bile ducts in the human liver is promoted by the CCR6/CCL20 axis [67]. Moreover, another study demonstrates the activation of the CCR6/CCL20 pathway in patients with chronic liver diseases and murine hepatic fibrosis [68]. 

### 4.4. Pancreas Diseases

In a study with a NOD model mouse of type 1 diabetes treated with resveratrol to prevent and treat type 1 diabetes, it was discovered that the expression of CCR6 decreased and the presence of CCR6^+^ IL-17 producing cells decreased in the pancreas after treatment. This study suggests that inhibition of those cells’ CCR6^+^ migration may provide an approach for treating type 1 diabetes [73]. CCR6 has also been related to chronic pancreatitis in a single-cell sequencing analysis with pancreatic immune cells. Single-cell sequencing revealed that the CCR6/CCL20 axis is upregulated in hereditary chronic pancreatitis when compared with idiopathic chronic pancreatitis; therefore, this signaling pathway could be a potential target in human hereditary chronic pancreatitis [69].

### 4.5. Dry Eye Disease (DED)

Dry eye disease (DED) is an immunoinflammatory disorder, and several studies implicate Th17 cells and the CCR6 receptor with pathogenic effectors in the disease [74,75]. Dohlman et al. studied the implication of the CCR6/CCL20 axis and Th17 cells infiltration on the ocular surface in DED and demonstrated that Th17 cells preferentially express CCR6 in the disease and CCL20 expression is upregulated at the ocular surface. The results suggested that the disruption of the axis could be a novel therapeutic approach to treating this disorder [74]. 

### 4.6. Endometriosis (EM)

Endometriosis is a chronic inflammatory gynecological disorder, and the CCR6/CCL20 system seems to be involved with the disease pathology. The inflammatory milieu of the tissue may present increased CCL20 expression and, consequently, increase Th17 migration to the endometriotic tissue and development of endometriosis by pro-inflammatory cytokines secreted by those cells [76]. A recent study related endometriotic stromal cells (ESCs) proliferation and migration with the CCR6/CCL20 axis and, thus, the pathogenesis of EM [77].

### 4.7. Renal Inflammation

The role of CCR6 in renal inflammation is not fully understood, but CCR6 and CCL20 might play a role in the recruitment of T and B cells to organize nodular infiltrates in chronic renal inflammation [78]. Indeed, it has been shown that CCL20 is upregulated in experimental glomerulonephritis in mice, resulting in CCR6-mediated T cell recruitment followed by renal tissue injury, albuminuria and loss of renal function [79]. 

### 4.8. Viral Infection

There is evidence of the critical role of the CCR6/CCL20 axis in HIV infection, supporting the need for early intervention to block HIV infection of CCR6^+^ T cells [81]. The CCR6/CCL20 axis helps propagate the virus to other body sites by recruiting Th17 and dendritic cells. Several studies have suggested that CCR6 has the ability to facilitate viral entry and replication; therefore, the receptor may act as a weak co-receptor of the viral entry [80]. As a result, the blockade of the CCR6/CCL20 mechanism may prevent the dissemination of HIV to key immunological sites.

CCR6 also plays a role in coronavirus disease (COVID-19), an infection caused by SARS-CoV-2 virus. There is an enrichment of CCR6^+^ CD8^+^ T cells in the lungs of mechanically ventilated patients and increased levels of CCL20 produced by SARS-CoV-2 infected alveolar or inflammatory macrophages. The CCR6/CCL20 axis drives T cell recruitment to the lungs and plays a role in sustained alveolar inflammation [72].

### 4.9. Inflammatory Bowel Disease (IBD)

Inflammatory bowel diseases affect the colon and the distal small intestine and are characterized by chronic intestinal inflammation. IBD comprises two phenotypes: ulcerative colitis and Crohn’s disease. There is a study in mouse IBD models in which the CCR6-CCL20 pair has a role in the generation of immune responses in the intestinal mucosa [82]. CCR6-deficient mice expressing spontaneous colitis displayed increased resistance to colonic inflammation reducing colitis severity in the mouse model [83]. The CCR6/CCL20 pathway has a critical role in the control of the immune response in the intestine. Although more studies are necessary, the axis could be a potential therapeutic target in the treatment of IBD [84,85].

### 4.10. Autoimmune Diseases

Considerable attention has been given to CCR6 in the pathology of several autoimmune diseases, including psoriasis, multiple sclerosis (MS), vitiligo, rheumatoid arthritis (RA) and atopic dermatitis (AD).

A significant up-regulation of CCR6 expression has been observed in Treg cells in the central nervous system (CNS), blood, spleen and lymph nodes cells in mice with experimental autoimmune encephalomyelitis (EAE), a mouse model of multiple sclerosis. The administration of Treg cells with blocked CCR6 to these animals supposedly ameliorates the disease [47]. Reboldi et al. reported that the CCR6/CCL20 axis is involved in initiating brain inflammation, and CCR6-knockout mice were highly resistant to EAE induction [89]. Therefore, CCR6 blocks could have potential therapeutic use in multiple sclerosis.

Rheumatoid arthritis (RA) is a severe chronic systemic inflammatory disease that causes joint destruction, and CCR6 expression contributes to Th17 cell function in the disease [96]. In inflamed RA joints, the secretion of CCL20 by myeloid cells is upregulated, and these cytokines promote the recruitment of CCR6^+^ Th17 cells. Moreover, CCL20 is produced by fibroblast-like synoviocytes contributing to local inflammation by inducing the recruitment of Th17 cells in the synovium [95]. In genome-wide association studies, CCR6 is associated with RA and could be an interesting candidate for a therapeutic approach [97,98]. 

Psoriasis is an immune-mediated chronic inflammatory skin disorder. CCR6+ T cells and CCL20 are abundant in the psoriatic skin of human psoriasis and experimental psoriasiform dermatitis [24,29]. The axis formed by TNF-α/IL-23/IL-17A plays a critical role in the pathogenesis of psoriasis. Several studies have reported that the CCR6/CCL20 axis is critically involved in the maintenance of the TNF-α/IL-23/IL-17A axis [92,93,94]. Therefore, the CCR6/CCL20 axis may be a promising target for treating psoriasis.

Higher percentages of CCR6+ T helper cells have been found in patients with systemic lupus erythematosus (SLE), especially in lupus nephritis. The receptor expression is correlated with disease activity and serological markers of disease severity [86,87]. However, more assays are still needed to determine the role of the CCR6/CCL20 axis in SLE.

## 5. CCR6 Blocking Antibodies and Therapeutic Approaches

Despite being one of the largest classes of proteins, most GPCRs remain drug-free. There are 398 GPCRs potentially druggable, and 325 of these receptors remain without drugs against them [102]. Traditional strategies are based on the inhibition of chemokine signaling by small molecules or peptide antagonists. Although CCR6 is a potentially druggable target, it is not easily amenable to the inhibitory effects of small molecules due to the difficulty in small molecule screens [103]. Moreover, the limited diversity of compound libraries complicates the discovery of new drugs. Therefore, other therapeutic approaches must be explored, such as neutralizing antibodies. Several monoclonal antibodies, small molecules and peptides CCR6 targeting are being developed, but, up to now, no research article has described CCR6 antagonists or inhibitors for clinical use [104]. Antagonists against CCR6 remain relatively rare in pipelines of biotech and pharmaceutical companies. Only a few antagonists against CCR6 have been identified (Table 3), and none of these compounds have been approved for the treatment of diseases associated with the CCR6/CCL20 axis [105]. 

Tawaraishi et al. identified 1,4-trans-1-benzenesulfonyl-4-aminocyclohexanes as human CCR6 inhibitors. They reported a picolinamide derivative, compound 35, that is a 1,4-trans-cyclohexane derivative that potently inhibited human B cell migration and ERK phosphorylation induced by CCL20, in a concentration-dependent manner, in human primary cell assays [106]. This compound could work effectively in vitro with an IC_50_ value of around 10 nM; however, there are no in vivo assays with this compound.

Another CCR6 antagonist compound to treat IBDs, named 1b, has been reported by Martina et al.; however, this work is at an early stage, and further studies on the optimization of potency and selectivity for CCR6 of the compound are currently ongoing [105].

Chemocentryx, recently acquired by Amgen, has developed several CCR6 antagonists, but none of these compounds have been approved for clinical use to date. In 2013, Chemocentryx was the first company in to identify a small molecule antagonist against CCR6, named CCX587. This oral inhibitor was developed to treat Th17-driven diseases in the skin, such as psoriasis, and may also treat rheumatoid arthritis [107]. Chemocentryx has also developed the small molecule, CCX9664, that blocks CCR6-mediated chemotaxis with an IC_50_ of 24 nM [108]. Another small molecule antagonist against CCR6 discovered by Chemocentryx is CCX2553, which has been shown to ameliorate inflammation in murine models of psoriasis [109]. The small molecule CCX624 (targeting CCR6 and CXCR2) reduced the T cells, neutrophils and inflammatory dendritic cell infiltrates and inflammatory symptoms [110]. The antagonist significantly alleviates the imiquimod-induced and IL-36α-induced murine psoriasiform inflammation [29]. All molecules presented promising results in preclinical models but have not yet been associated with a scientific publication or beneficial results in clinical trials [25]. 

Pfizer and Sosei Heptares have a small molecule, PF-07054894, the antagonist of CCR6, that is in a Phase 1 clinical trial (ClinicalTrial.gov: NCT04388878). PF-07054894 is an antagonist targeting inflammatory bowel disease. The purpose of the study is to assess the safety, tolerability and pharmacokinetics of single and multiple ascending oral doses of the compound in healthy adult participants; however, there are still no results from this clinical trial [114]. 

It is noted that three antibodies against CCR6 have been patented by Pharma Companies (WO2013184218A1, WO2013005649A1 and WO2016059253A1). In the patent WO2013184218A1 (Msm Protein Technologies, Waltham, MA, USA), Kim et al. developed an antagonist antibody from phage display human antibody libraries selected against human CCR6-presenting liposomes [111]. In the patent WO2013005649A1 (Kyowa Hakko Kirin, Tokyo, Japan), Yuya et al. obtained a neutralizing antibody from SD rats immunized with CHO cells expressing human CCR6 [112]. More recently, in the patent WO2016059253A1 (Glenmark Pharmaceuticals, Mumbai, India), Lissilaa et al. developed antibodies that bind to CCR6, reducing ligand–receptor binding [113]. All such prior antibodies do not have the properties necessary to be suitable as therapeutic antibodies. 

There are several commercially available anti-human CCR6 monoclonal antibodies, such as 53103 (R&D Systems, Minneapolis, MN, USA), 11A9 (BD Biosciences, Franklin Lakes, NJ, USA), 4C6 (ATGen, Montevideo, Uruguay), R6H1 (eBioscience, San Diego, CA, USA), G034E3 (BioLegend, San Diego, CA, USA) or MM0066-3L1 (abcam, Cambridge, UK). Nevertheless, they are for research use only, and no therapeutic approaches have been reported for these antibodies.

Disruption of the CCR6/CCL20 axis could include CCR6 and CCL20 inhibitors. CCL20 is the high-affinity ligand of CCR6, but low-affinity binding of human beta-defensin-1 and -2 to the CCR6 receptor have been reported. Although hBD has a lower affinity for CCR6, as compared with CCL20, both ligands compete for binding to their receptor and. Thus, a CCL20 blocking antibody is not enough to completely inhibit CCR6 function [6,42]. Blocking the receptor directly with an anti-CCR6 antibody would be a more appropriate strategy, increasing the possibilities of neutralizing both high- and low-affinity ligands, not only CCL20. 

Th17 cells are recruited through the CCR6/CCL20 axis and release the pro-inflammatory IL-17 cytokine in damaged tissues promoting inflammation. Thus, another therapeutic approach for treating the diseases in which CCR6/CCL20 signaling is implicated is the development of inhibitors against IL-17. However, inhibition of CCR6 may be more feasible and effective than inhibiting IL-17 from a drug development perspective because IL-17 has many isoforms, and it is difficult to inhibit all of them with a single inhibitor [103]. 

Although small molecules still dominate the agents targeting GPCRs, the proportion of these drugs has declined since 2010 [4]. Therapeutic antibodies represent a promising alternative to these conventional therapeutic agents in drug discovery. Compared with chemical drugs, mAbs have obvious advantages of improved specificity, affinity and other pharmacological properties and are being developed against cancer, inflammation and metabolic disorders [5,115,116]. Many drugs’ cross-reactivities among GPCRs subtypes could cause undesired side effects. Compared with small molecules and peptide drugs, therapeutic antibodies have much-reduced side effects probably due to the better specificity mAbs [4,116,117]. A limitation in the development of the CCR6/CCL20 axis disruptors is the lack of X-Ray structure of CCR6 for in silico drug design, and only recently has been published a cryo-electron microscopy (cryo-EM) structure of the human CCR6 bound to CCL20 ligand and Go protein [8]. This structure can be used to obtain relevant insights into the mechanism of activation of CCR6. The representations of CCR6 available in the GPCR database can also be used to map common hotspots for ligand binding or receptor selectivity and to delineate specific CCR6/CCL20 interactions [21]. 

The attempts to obtain drugs against CCR6 could be unsuccessful due to the shortage of compound availability and the insufficient diversity of small molecules that difficult the ligands screens. Moreover, the lack of relevant structural information for drug design results in drugs with unfavorable pharmacokinetic and pharmacodynamic properties that do not progress in clinical evaluation. The drugs discovered have low binding affinity or potency and do not have antagonistic activity. Thus, no therapeutic approach has been reported for the antibodies and small molecules obtained to date.

Although CCR6 has long been considered a highly desirable target for drug discovery purposes, only a few small molecules and antibodies antagonists have been reported; none of these have been approved for treating diseases linked to the CCR6/CCL20 axis. Therefore, CCR6 could be a therapeutic target to develop therapeutic antibodies for treating the diseases in which CCR6/CCL20 signaling is implicated.

## 6. Future Perspectives

As we pore over the research involving the discovery of drugs against CCR6, we can find the development of ten inhibitor agents in the last ten years. Most were small molecules, and only three were monoclonal antibodies; therefore, drugs based on antibodies are poorly exploited and should be further investigated. The small molecules libraries have insufficient diversity, and the use of these drugs have been unsuccessful due to the few pharmacological properties. Attempts have been made to treat CCR6/CCL20 axis-related conditions without focusing on the highlight of these drugs’ development restrictions, which are limitations of potency, selectivity and bioavailability. To better approach the CCR6/CCL20 axis-related disorders, innovative drugs are needed, such as therapeutics antibodies, which have high affinity and specificity for the target. Therapeutic antibodies could open new ways to test more effective therapeutic interventions to treat CCR6-related diseases in the future. 

Based on the current knowledge, strategies and rationales in developing therapeutics antibodies against CCR6 should consider the following information: (1) Current drugs based on small molecules have limitations of drug properties. (2) Small molecules libraries are lacking in diversity. (3) Screening of small molecules is very complicated. (4) Artificial intelligence drug design through the structural biology (cryo-electron microscopy) approach makes it possible to design new inhibitors. (5) Therapeutic antibodies have high affinity and specificity to their targets. (6) Further optimizations can be undertaken in vitro to improve antibody properties. (7) Repurposing existing mAbs is an opportunity for accelerating and optimizing drug development. 

In summary, CCR6 is a target with a high therapeutic potential to treat inflammatory diseases. Many reports have been published about the development of unsuccessful inhibitors against CCR6; however, with the advance in novel technologies and high-resolution structures of CCR6, the discovery of therapeutic antibodies against CCR6 could be considerably encouraged.

## 7. Conclusions

CCR6 is a potential target in treating various diseases, such as cancer, inflammatory and autoimmune disorders. The CCR6/CCL20 axis plays a critical role in maintaining immune homeostasis. CCR6 is involved in the recruitment of Th17 and Treg cells, and recruited Th17 cells release pro-inflammatory cytokines in damaged tissues, promoting inflammation. The imbalance of the Th17/Treg ratio is implicated in numerous diseases. A strategy to be considered to develop drugs against CCR6 may be the inhibition of migration of CCR6^+^ cells with antagonist antibodies. In vitro and preclinical studies show that inhibition of CCR6 could provide a powerful tool in the treatment of CCR6 diseases. There is a lack of compounds approved against the receptor, and small molecule inhibitors have not advanced in clinical trials. Therapeutic antibodies are effective as an alternative to small molecule drugs, and these emerging agents could therefore provide valuable new treatment options to the CCR6 target. The data so far indicate that CCR6 is a valuable target for developing therapeutic monoclonal antibodies for treating CCR6/CCL20 axis-related diseases.

## Figures and Tables

**Table 1:** CCR6^+^ cells.

Cell Type	Reference
B cell	[19,22,23,24]
Immature DC	[19,22,24,25]
ICL-3	[19,22,25]
Langerhans cell	[26,27]
NK cell	[17,28]
NKT cell	[19,22,26]
Neutrophils	[19,22]
Th9 cell	[28]
Th17 cell	[19,22,23,29]
Th22 cell	[28,30]
Treg cell	[19,22,23,24]
γδT cell	[28]

DC—dendritic cell; ILC-3—innate lymphoid cell 3; NK cell—natural killer cell; NKT cell—natural killer T cell; Th cell—T helper cell; Treg cell—regulatory T cell.

**Table 2:** CCR6-related diseases.

Disease	Reference
Asthma	[26,50,51]
Atopic dermatitis	[52,53]
Cancer	[23,54,55,56,57,58,59,60,61,62,63,64,65,66]
Cholestatic liver diseases	[67]
Chronic liver diseases	[68]
Chronic pancreatitis	[69]
COPD	[70,71]
COVID-19	[72]
Diabetes	[73]
DED	[74,75]
Endometriosis	[76,77]
Glomerulonephritis	[78,79]
HIV	[80,81]
IBD	[22,26,82,83,84,85]
Systemic Lupus erythematosus	[86,87]
Multiple sclerosis	[25,47,88,89]
Psoriasis	[24,29,90,91,92,93,94]
Rheumatoid arthritis	[25,95,96,97,98]
Vitiligo	[99]

COPD—chronic obstructive pulmonary disease; COVID-19—coronavirus disease 2019; DED—dry eye disease; HIV—human immunodeficiency virus; IBD—inflammatory bowel disease.

**Table 3:** Inhibitors against CCR6.

Name	Company	Type of Inhibitor	Reference
Compound 35	Takeda Pharmaceutical	Small molecule	[106]
Compound 1b	MedChemExpress	Small molecule	[105]
CCX587	Chemocentryx	Small molecule	[107]
CCX9664	Chemocentryx	Small molecule	[108]
CCX2553	Chemocentryx	Small molecule	[109]
CCX624	Chemocentryx	Small molecule	[29,110]
WO2013184218A1	Msm Protein Technologies	Antibody	[111]
WO2013005649A1	Kyowa Hakko Kirin	Antibody	[112]
WO2016059253A1	Glenmark Pharmaceuticals	Antibody	[113]
PF-07054894	Pfizer	Small molecule	[114]

## Data Availability

No new data were created or analyzed in this study. Data sharing is not applicable to this article.
